# The Use of Telegram in Surgical Education: Exploratory Study

**DOI:** 10.2196/35983

**Published:** 2022-09-27

**Authors:** Marcus Khai Siang Soon, Laura Martinengo, Junde Lu, Lorainne Tudor Car, Clement Luck Khng Chia

**Affiliations:** 1 Lee Kong Chian School of Medicine Nanyang Technological University Singapore Singapore; 2 Department of General Surgery Khoo Teck Puat Hospital Singapore Singapore; 3 Department of Primary Care and Public Health School of Public Health Imperial College London London United Kingdom; 4 Yong Loo Lin School of Medicine National University of Singapore Singapore Singapore

**Keywords:** COVID-19, undergraduate medical education, distance education, social media, Telegram, general surgery, messaging apps

## Abstract

**Background:**

The COVID-19 pandemic has disrupted medical education, shifting learning online. Social media platforms, including messaging apps, are well integrated into medical education. However, Telegram’s role in medical education remains relatively unexplored.

**Objective:**

This study aims to explore the perceptions of medical students regarding the role of messaging apps in medical education and their experience of using Telegram for surgical education.

**Methods:**

A Telegram channel “Telegram Education for Surgery Learning and Application (TESLA)” was created to supplement medical students’ learning. We invited 13 medical students who joined the TESLA channel for at least a month to participate in individual semistructured interviews. Interviews were conducted via videoconferencing using an interview guide and were then transcribed and analyzed by 2 researchers using inductive thematic content analysis.

**Results:**

Two themes were identified: (1) learning as a medical student and (2) the role of mobile learning (mLearning) in medical education. Students shared that pandemic-related safety measures, such as reduced clinic allocations and the inability to cross between wards, led to a decrease in clinical exposure. Mobile apps, which included proprietary study apps and messaging apps, were increasingly used by students to aid their learning. Students favored Telegram over other messaging apps and reported the development of TESLA as beneficial, particularly for revision and increasing knowledge.

**Conclusions:**

The use of apps for medical education increased during the COVID-19 pandemic. Medical students commonly used apps to consolidate their learning and revise examination topics. They found TESLA useful, relevant, and trustworthy.

## Introduction

COVID-19 was declared a global health emergency on January 30, 2020 [1]. Consequently, medical education was disrupted due to the social distancing efforts to contain disease spread and transmission [2-4]. Clinical-year students were particularly affected as clinical rotations were suspended or altered [2] or the students were deployed to perform clinical tasks [5]. To compensate for the lack of face-to-face interactions, medical schools increasingly leveraged the use of digital technologies [2] as students and educators were required to quickly adapt to virtual learning environments, such as videoconferencing and social media platforms, websites, blogs, or other educational materials available online [4]. The use of digital technologies in education has increased steadily over the past decade, including podcasts and videos with flipped classrooms, mobile devices with apps, video games, simulations (part-time trainers, integrated simulators, virtual reality), and wearable devices [6].

Digital education, defined as “the act of teaching and learning by means of digital technologies” [7], is an encompassing term including a wide variety of teaching methods from digital books to complex technology, such as virtual reality. Mobile learning, or mLearning, constitutes 1 of several digital education modalities, and it is defined as “learning across multiple contexts, through social and content interactions, using personal electronic devices” [8] and appears effective in improving the knowledge and skills of students and professionals in health care [9].

Social media refers to websites or apps that “allow for the creation and exchange of content generated by users” [10]. These include collaborative projects or wikis, blogs, content-sharing sites, networking platforms, social games [10], and, often, messaging apps. Social media platforms have long been well integrated into medical education [11-17], and they may be especially appealing to younger students [16] as they are readily accessible from smartphones [17], easy to use [16], and affordable [12,16,17]. The use of social media platforms in medical education has been associated with improved knowledge (examination scores), attitudes (empathy), and skills (reflective writing) [18]. They also promote student collaboration [10,12,16-18], learner engagement [16,18], feedback [16,18], and professional development [18] and are generally well accepted by students [19,20].

Messaging apps, particularly WhatsApp, are being increasingly used in medical education [21-23]. Alternatively, the use of Telegram, a free, cross-platform, cloud-based messaging app [24] popular with younger individuals, remains relatively unexplored [11]. Several Telegram features, such as large group chats, broadcast channels to reach large audiences, and polls, may facilitate access to educational resources and offer unlimited sharing capacity and collaborative peer learning, while providing heightened security [11].

The increased digitalization of medical education, greatly enhanced during the heightened social distancing measures to contain COVID-19, supports further evaluation of the use of Telegram to assist learning in medical schools. Therefore, in this study, a Telegram channel “Telegram Education for Surgery Learning and Application (TESLA)” was developed to support general surgery learning by offering regular access to multiple-choice questions (MCQs). The acceptability of the Telegram channel among students was subsequently assessed by conducting in-depth interviews to explore student’ views of the channel and, more generally, of the use of mobile messaging apps to support learning in medical schools. This study aims to explore the perceptions of medical students regarding the role of messaging apps in medical education and their experience of using TESLA for surgical education.

## Methods

### Study Design and Setting

This qualitative study, including semistructured interviews, was a collaboration between the Department of General Surgery in Khoo Teck Puat Hospital and the Lee Kong Chian School of Medicine (LKCMedicine) in Singapore. The study was conducted between August and September 2021.

### Ethical Considerations

Ethical approval was obtained from the Nanyang Technological University Institutional Review Board (#IRB-2021-377).

### Telegram Channel to Support Surgical Education

**Figure 1:**
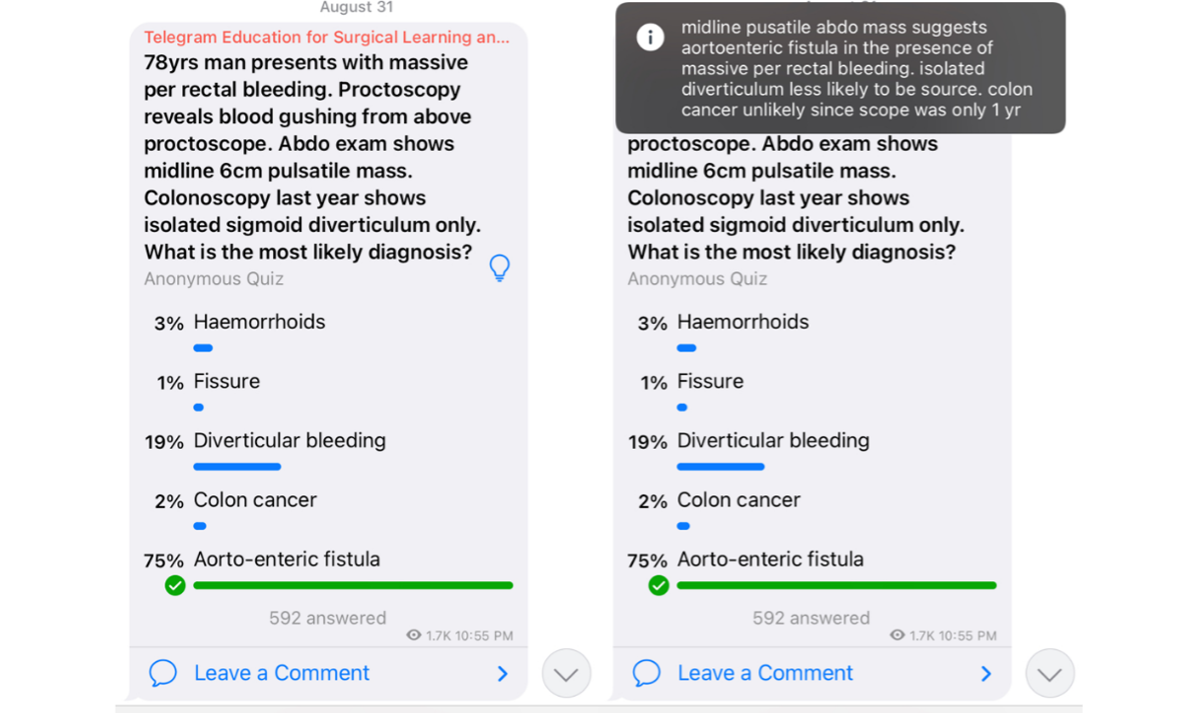
Screenshot of an MCQ (left) with its explanation (right). MCQ: multiple-choice question.

TESLA was developed in October 2020 to support students’ learning of general surgery during the COVID-19 pandemic. The questions were constructed using clinical scenarios encountered during clinical practice by a practicing general surgeon (author CLK). The channel consisted of weekly MCQs, succinct explanations of the correct responses aligned with Telegram word limit restrictions, and supplemental learning resources, such as illustrations and relevant published papers (Figure 1). Students answered the questions anonymously and were encouraged to leave comments or request clarifications via a group chat function. This format appears to benefit students by promoting learning and enabling information retention [25,26].

### Participants and Recruitment

Medical students attending clinical years 4 and 5 in LKCMedicine who had completed their general surgery module and had used TESLA for at least 1 month were invited to participate in this study. Per the qualitative research methodology, the sample size was expected to be 10-12 participants [27], according to reaching data saturation, defined as no new themes emerging in 3 consecutive interviews [28]. All participants read a study information sheet and signed the informed consent form before data collection began. After completing the interviews, participants were compensated with a digital voucher of SG $20 (US $14.22).

### Development of the Interview Topic Guide

A thorough literature review was conducted to develop an inventory of open- and closed-ended questions. The initial interview topic guide was pilot-tested on 3 LKCMedicine students and reviewed to improve the clarity and flow of the interview (Multimedia Appendix 1). The interview included questions about the impact of COVID-19 on medical education, the use of apps to support learning, and the use of the TESLA channel.

### Data Collection and Analysis

Individual, online semistructured interviews were conducted by a male undergraduate year 4 medical student (author MS) using Zoom, a videoconferencing app. MS took part in qualitative research training before conducting the interviews. Interviews lasted around 30 minutes and were audio-recorded using Zoom. The audio recordings were transcribed and proofread (MS) to ensure accuracy. Participants remained anonymous in the transcription and data analysis. The data were analyzed following Burnard’s thematic analysis method [29], comprising familiarization with the transcripts by reading them multiple times, creation of initial codes, and subsequent development of themes from the codes by 2 independent researchers. The codes were subsequently compared and discussed by the researchers to reach a consensus on the final list of codes (Multimedia Appendix 2). We used NVivo (QSR International), a computer-assisted qualitative data analysis software package, to analyze the data. The study was reported according to the Consolidated Criteria for Reporting Qualitative Research (COREQ) guidelines (Multimedia Appendix 3) [30].

## Results

### Participants and Themes

We interviewed 13 students for this study, of which 11 (85%) were in year 4 and 2 (15%) were in year 5. Their participation in the surgical education support channel ranged from 1.5 to 18 months.

**Figure 2:**
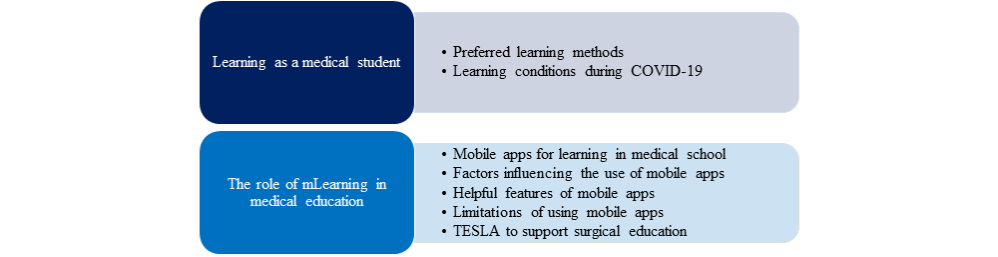
Themes and subthemes arising from thematic analysis of the interview transcripts. mLearning: mobile learning; TESLA: Telegram Education for Surgery Learning and Application.

Two main themes were identified: (1) learning as a medical student (2) the role of mLearning in medical education (Figure 2). We present each theme, along with its subthemes and relevant participants’ quotes, next.

### Theme 1: Learning as a Medical Student

#### Preferred Learning Methods

Students valued hands-on, experiential learning as they felt that seeing actual patients is more conducive to learning.

Real learning comes from seeing the real signs and how the patients present, as well as talking to patients in the wards.

Participant 3 (P3)

I think it is a little bit different between digital and physical, in a sense that you do not get to feel it or ask questions immediately if you do not understand.

P4

Particularly, students considered that the learning of skills should always occur in a face-to-face setting that allows students to practice the skills while receiving real-time feedback from mentors.

…The best way to learn these skills will be with a clinical mentor who can see and give you personalized feedback.

P2

…Skills need to be practiced and seen in person…

P5

#### Learning Conditions During COVID-19

The social distancing measures associated with COVID-19 impacted traditional learning practices, particularly the interaction with patients. All students reported increased restrictions in the hospitals, such as reduced clinic allocations and the inability to visit wards.

We could not really go around the wards as freely anymore. We also could not see a lot of patients, so the patient load was decreased as well. Yeah, we had restrictions in the OT, and we cannot have too many people around when having bedside tutorials.

P1

What I lost in the pandemic due to the restrictions is not the knowledge, but the practical skills and the experiences of being immersed in the different situations.

P11

This resulted in decreased exposure to patients and a shift of a significant portion of learning online.

I think it was affected because our postings were cut short and a lot of lessons were moved online.

P5

…You cannot go to the hospital anymore, and then you have to attend lectures online,…you do not get to see the clinical presentations.

P13

Given these changes during the pandemic, most students found their clinical and surgical rotations substantially shorten.

…Since ward time has been reduced, I think there has also been a reduction in content delivery, like bedside tutorials.

P4

I think a lot of like opportunities were reduced and there were a lot more restrictions. So, it was more difficult to get practical hands-on learning.

P11

### Theme 2: The Role of mLearning in Medical Education

#### Mobile Apps for Learning in Medical School

Many students used apps to aid their learning, including proprietary study apps and messaging apps. The study apps consisted of question banks and flashcards (Amboss, Anki, Qstream, Capsule, and Osmosis) and an evidence-based clinical resource tool (UpToDate). These apps allowed students to review previously learned topics leveraging study techniques such as spaced repetition and active recall.

For educational videos, I use the internet, YouTube, Osmosis, and Amboss. For knowledge and practice questions, I use the internet, Telegram, and also other educational apps, such as the flashcard app Anki.

P3

My school uses Qstream, which also prompts questions for [you] to answer...There are also other applications that I use, such as Amboss, which also does the same. I also use Anki.

P9

Students also used messaging apps for their learning, particularly Telegram, WhatsApp, and Instagram. Students were generally familiar with these apps as they used them for social communications with family and friends. These apps were often used to communicate with tutors and peers, as well as to join specific channels offering study support tools.

I use the Telegram channels as well as polls on Instagram stories.

P9

This Telegram group, some Instagram pages that our batchmates created, online notes and also YouTube.

P12

Messaging apps were also used for communication with friends and family members. Among these apps, Telegram was favored for its friendlier user interface and multifunctionality.

Telegram is good because it has more features and has more stickers as well.

P5

I prefer Telegram because I think it is more user-friendly than WhatsApp and it is easier to navigate.

P13

Students were aware that the use of mobile apps would not replace more traditional ways of learning, for example, textbooks, lectures, and, particularly, patient interactions.

…It works to complement the main learning from, like, textbooks or seniors notes, etc, and to reinforce whatever I have already learnt…

P2

Because it is virtual, there is a lack of face-to-face interaction and the ability to perform physical examination or take a proper face-to-face history. I think that is a big issue that people feel with regards to personnel competency in medicine…I think it is something that no amount of virtual learning can replace.

P4

…Mobile-based education is more for contents, while clinical skills have to come through the clinical context.

P9

#### Factors Influencing the Use of Mobile Apps

In general, students considered using apps that were free of charge or affordable.

For some mobile apps like Amboss, our school actually helped to pay for their subscription fees and so made it available for all of us to use.

P2

They also favored apps if they were considered useful or entertaining.

If I think I learnt quite a lot from it or I feel that their way of teaching is effective, then I will use it more.

P2

Students also considered using apps if they were recommended by their peers or seniors.

Even my seniors who went through the rotations without the pandemic were already using them and recommended them to me.

P1

It's more of a recommendation by word of mouth, like whether this particular app is good or not.

P2

Students appreciated the convenience of accessing mobile apps at any time and place.

These apps make learning accessible and convenient since we can study anytime and anywhere, even when queueing up for food or when traveling between places.

P1

The greatest pull factor is how convenient it is to use the phone. I can easily use these apps when I am on public transport.

P3

#### Helpful Features of Mobile Apps

The apps offered a wealth of resources and information that motivated students to learn.

The number of questions available on these question banks online is really a lot.

P5

Another pro is that it stimulates active recall.

P6

Furthermore, messaging apps facilitated 2-way communication with tutors and peers.

I think in my school, there are, like, a few groups that me and my friends created, just to, like, practice taking histories from each other or, like, trying to identify what is an important topic and what is high yield to learn.

P2

I also use it to communicate with friends during postings and to receive posting information from official sources.

P11

#### Limitations of Using Mobile Apps

Barriers to the use of study apps included high subscription costs and hardware limitations, such as small phone screens, battery drainage, and an inflexible typing keyboard.

If I use my phone too much, the battery will also run out very quickly.

P1

Some apps require subscriptions or a one-time payment for use. So, such costs are like a barrier if you do not like to or do not wish to pay for these apps.

P2

It is harder for me to edit things because the phone keyboard is less versatile.

P3

Other limitations were associated with accessing apps using a phone. These included limited content on display, the information presented in an unorganized manner, and greater difficulty taking notes.

…It is very difficult to search for the different files because they will be, like, mixed with other non-work or [non]-education-related chats, which is very messy.

P1

It is also harder to take notes…It is harder to refer to notes or huge chunks of texts on the train as compared to when at home.

P10

An issue that I encounter with mobile learning, which also exists for hard-copy learning, is that explanations may not be complete.

P11

When using study apps, students felt that the information was sometimes irrelevant to the local context:

The conditions and presenting complaints are very localized to their countries. For example, in America, they have certain diseases that are more prevalent and, to them, they are a must-know. But then to us, it is something that we were taught not to pay so much attention to.

P13

Alternatively, the use of messaging apps could potentially be intrusive with notification alerts and blurring of lines between personal use as a communication tool versus a tool for learning.

I don't like it, because questions and work messages get mixed together in one app and then it kind of distracts the learning.

P1

#### TESLA to Support Surgical Education

In general, students expressed positive feedback toward the development of the mLearning platform, which they considered useful to consolidate and maybe augment their learning and review before tests.

I think it is quite useful because of the case-based style of questions. The question difficulty is appropriate…

P3

The Telegram group is definitely a very useful tool to augment learning. Any form of revision questions will definitely help students in clarifying questions.

P10

Particularly, students appreciated that the resources were developed by the teaching staff of the Department of Surgery.

The information in the group is provided by doctors, and so, the information is more credible. Compared to the other groups, I would be more inclined to trust the information from this group.

P2

Since it is a channel created by a doctor, it makes the questions more legitimate as compared to if being made by students.

P10

Students considered that the questions, and the associated supplementary material, were clinically relevant, of appropriate difficulty, and relevant to the local context. They also valued that responses to the channel questions were anonymous, an aspect that allowed students to attempt the task without the stress of being wrong.

…The content posted is very relevant to the Singapore medical student, and it is the important stuff that you cannot miss.

P3

Some of the pictures are what students might not get the opportunity to see. So, I think that is good.

P5

When it is an anonymous poll, it takes away any shame in answering the questions wrongly. Even if I do not know, I can just give it a shot and see how I am doing.

P9

Alternatively, the associated discussion forum required the students’ identities to be disclosed, and this feature was a major deterrent to students clarifying their doubts.

I think it is weird that everyone in the group will be reading your question. So, I guess, maybe one thing that I would prefer is to ask questions anonymously.

P1

It seems that no one uses the comment feature. Probably it is because it is not anonymous, and people are shy to use it even if they have questions.

P10

Students reported that the questions were presented without following specific themes or surgical specialties. Some questions lacked explanations, or if present, they were brief and difficult to comprehend. Students were aware that this may be an inherent Telegram limitation, and they suggested that providing links to extra information, as well as categorizing the content, would improve the channel.

I feel that the Telegram group is a bit messy as the questions are random...Telegram is not able to provide such clear classification of the questions, and a new learner might feel that their learning would be all over the place…The explanations are very short since there is a word limit to the questions and explanations. Hence sometimes, I do not fully understand the explanation, because it's not complete.

P1

I think the explanations to the questions are quite short. Hence, I think there is room to expand on that, for instance, providing resources for students on where to find articles or guidelines. Maybe, the doctors can also explain their approach to solving the questions. It would help lead to a more comprehensive understanding of the explanations.

P12

## Discussion

### Principal Findings

This study explored medical students’ perceptions of learning during the COVID-19 pandemic, the role of mLearning in medical education, and the use of a Telegram channel to support surgical education. Students experienced important changes in their learning during the COVID-19 pandemic, particularly the substantial decrease in clinical, hands-on, and experiential learning, leading to a rise in virtual learning. The use of mobile apps to support learning, although already in use prepandemic, increased. Students found these apps useful as a refresher or to consolidate learning and valued the development of the TESLA, as they found it relevant and trustworthy.

Medical students consistently reported disruptions in learning due to the pandemic, particularly the substantial decrease in face-to-face, experiential learning in health care institutions. Digital learning tools, including mobile apps, were increasingly used to compensate for the lack of face-to-face learning. This shift to digital-based learning was not unique to our student population but was consistently reported in medical schools worldwide [5,31,32]. Digital tools were an adequate substitute for theoretical learning and revisions ahead of examinations, although they could not substitute face-to-face patient interactions in the learning of clinical skills. The use of digital technologies in medical education has steadily increased for over a decade, although their adoption was not consistent. However, only with the onset of the COVID-19 pandemic was widespread adoption of digital learning seen in medical schools worldwide. This educational shift was accompanied by added flexibility and increased emphasis on individual learning preferences, which are valued by students and educators alike. At the same, time, the wide differences in digital readiness observed in high- and low- and middle-income countries translated to inadequate training for students unable to access educational materials [32,33], while the lack of access to health care institutions also impacted the acquisition of critical clinical skills [33].

Mobile apps to support medical education were widely used by students. They selected a combination of proprietary study apps, consisting mostly of question banks and other revision tools, and study groups created in popular messaging apps, such as the Telegram group presented in this paper. A recent systematic review on the use of mobile apps in education in health care professions reported these tools as effective to enhance knowledge and skills in a range of topics, including anatomy, dermatology, and surgery [34], but it did not provide information about the type of mobile apps described in the included studies. Furthermore, another review on the use of social media in medical education in the context of the COVID-19 pandemic highlighted the use of messaging apps, such as Facebook, Twitter, Instagram, and WhatsApp [35], to organize groups to post revision topics or questions and share articles and as communication tools to inform students of changes in schedules, important deadlines, or administrative tasks [15,18]. None of these studies presented compelling evidence that the use of proprietary study apps or messaging apps would be advantageous. Furthermore, the students interviewed for this study appear to use a combination of both types of apps, each of which may provide specific advantages or disadvantages. For example, study apps may offer students a trustworthy source of information, particularly if recommended by instructors or peers, but their content may not be relevant to the local context and the associated cost might be a deterrent for some students. In contrast, messaging apps are readily available, usually free of charge, and students may already be familiar with their functionalities, but their content may not be adequately verified.

The students interviewed in our study consistently reported a preference for Telegram over other messaging apps, referring to the flexibility and enhanced functionalities the app offers. The evidence for the use of social media and messaging apps has focused particularly on the use of WhatsApp, Facebook, and Instagram compared to Telegram, whose role in medical education remains relatively unexplored [11]. Telegram may offer key advantages, such as a poll function that allows for the development of MCQs, the possibility of creating large groups of up to 200,000 members, and enhanced data security safeguards, which may increase its appeal in educational settings [11,24]. Further studies on the use of Telegram for medical education are required.

### Strengths and Limitations

Our study was undertaken in 1 medical school in Singapore, a city-state with a high penetration of smartphones, and as such, our findings and recommendations may not be generalizable to other contexts. Although our findings were more focused on the use of Telegram channels, the findings may apply to the use of other messaging apps to support medical education.

We used a stringent qualitative methodology in this study. The number of students recruited was aligned with sample size guidelines for qualitative studies, and they had varied experiences with the use of mobile apps to support medical education. Data analysis was performed by 2 independent researchers and reviewed by other members of the research team to increase the validity of our findings.

### Implications for Future Work

The findings of this qualitative evaluation of medical students’ perceptions of the use of messaging apps appear to support their use in medical education. Table 1 presents a series of suggestions to implement when creating study groups using messaging apps, based on our experience deploying TESLA and students’ feedback. Nevertheless, experimental clinical trials evaluating the effectiveness of mLearning compared to other learning approaches are urgently needed.

**Table 1:** Suggested features to include when creating messaging app groups to support medical students’ learning.

Suggested features		Recommendations
**Delivery**		
	Recognizable	A catchy channel name
	Accessible	Clear guidance for users on how to join the study group
	Anonymity	Participants to be allowed to answer questions anonymously
	Timely	Questions to be posted during the academic year and not during holidays
	Regular	Questions to be posted at regular, predictable intervals
**Content**		
	Credible and trustworthy	Created by health professionals or reference to trusted information sources
	Organized	Study group content clearly organized, with adequate signposting to study topics, etc
	Clear and unambiguous	Indication of the difficulty level of each question Explanations provided for all options and all questions Links provided to learning resources, such as peer-reviewed papers, book articles, pictures/photos, short videos
	Relevant to the target population	Appropriate question difficulty Real-life clinical cases Contextualized to the local setting
**Format**		
	Type of questions	Variety of question formats, including MCQs^a^, open-ended questions, etc
	Engaging	Bite-sized information Case-based questions Variation of question difficulty Use of visuals
	Instant feedback	Immediate response after participant’s response Showing participant option selection rates

^a^MCQ: multiple-choice question.

### Conclusion

The use of apps and other digital tools to support medical education increased during the COVID-19 pandemic. mLearning was commonly used among medical students to consolidate their learning and revise examination topics. In general, Telegram was preferred over other messaging apps for its user interface and multifunctionality, and TESLA was evaluated as useful, relevant, and trustworthy. Experimental clinical trials on the use of mobile apps to support medical education are urgently needed.

## Appendix

Multimedia Appendix 1 Interview guide.

Multimedia Appendix 2 Codebook.

Multimedia Appendix 3 COREQ 32-item checklist.

## Abbreviations

LKCMedicine: Lee Kong Chian School of Medicine
MCQ: multiple-choice question
mLearning: mobile learning
TESLA: Telegram Education for Surgical Learning and Application
