# Supplementary material for: The Use of Telegram in Surgical Education: Exploratory Study
Source: JMIR Med Educ. 2022 Sep 27;8(3):e35983. doi: 10.2196/35983 (PMC9518707; doi:10.2196/35983)
Supplement: Multimedia Appendix 1 [file mededu_v8i3e35983_app1.docx]

**Appendix 1: Interview guide**

In this appendix, the list of interview questions and prompts used for data collection is shown.

Questions on demographics:

| No. | Questions |
| --- | --- |
| 1 | Which medical school are currently attending? |
| 2 | Which year of medical school are you currently in? |
| 3 | Have you completed your general surgery rotation? |
| 4 | Are you currently taking part in your general surgery rotation? |
| 5 | How long have you been using Telegram? |
| 6 | What do you primarily use Telegram for? |
| 7 | What other messaging apps do you use? |
| 8 | How long have you been participating in the General Surgery MedEd Telegram group? |

Questions on mobile learning and the General Surgery MedEd Telegram channel:

| No. | Questions | Prompts |
| --- | --- | --- |
| 1 | How did the Covid-19 pandemic affect your medical education? | Medical education in general; Surgical education |
| 2 | What role has mobile learning (i.e., learning delivered via mobile devices) had on your education? | During this pandemic; In general; In surgical education; In other areas; Types of mobile learning used; Pros/Cons to its use |
| 3 | What is your view on the use of messaging apps (e.g. Telegram or WhatsApp) in your medical education? | Areas in which they can be used; Pros/cons of using them; Preferences |
| 4 | What is your view of the General Surgery MedEd Telegram group? | Benefits/limitations |
| 5 | What impact did the General Surgery MedEd Telegram group have on your surgical competency? | Knowledge; Skills |
| 6 | What impact did the General Surgery MedEd Telegram have on your attitudes towards surgery? | Surgical training; Surgery as a speciality/choice of residency |
| 7 | What is your view of the potential inclusion of the General Surgery MedEd Telegram group in formal undergraduate surgical education in future? | Benefits/limitations |
| 8 | How can the General Surgery MedEd Telegram group be improved? | Additional content; Features |
| 9 | What is your view on other similar educational Telegram groups that you have used? | Similarities/differences; Advantages/disadvantages |
| 10 | What is your view on the use of such Telegram groups in other areas of your medical education? | Areas; Format of the channel; Advantages; Disadvantages |
| 11 | Are there any other observations or comments that you would like to share with me today? |  |
