# Supplementary material for: The Use of Telegram in Surgical Education: Exploratory Study
Source: JMIR Med Educ. 2022 Sep 27;8(3):e35983. doi: 10.2196/35983 (PMC9518707; doi:10.2196/35983)
Supplement: Multimedia Appendix 2 [file mededu_v8i3e35983_app2.docx]

**Appendix 2: Codebook**

In this appendix, the themes, sub-themes and codes generated from qualitative analysis are shown.

| Themes | Sub-themes | Codes | Quotes |
| --- | --- | --- | --- |
| Theme 1: Learning as a medical student | | | |
|  | Preferred learning method | Hands-on, experiential learning | P01 “For me, I still prefer something a bit more traditional.”  P03 “Real learning comes from seeing the real signs and how the patients present, as well as talking to patients in the wards.”  P04 “I think it is a little bit different between digital and physical, in a sense that you do not get to feel it or ask questions immediately if you do not understand.” |
|  |  | Face-to-face setting of learning | P02 “ … the best way to learn these skills will be with a clinical mentor who can see and give you personalized feedback.”  P05 “… skills need to be practised and seen in person…” |
|  |  |  |  |
|  | Learning conditions during COVID-19 | Increased restrictions in the hospitals | P01 “we could not really go around the wards as freely anymore. We also could not see a lot of patients, so the patient load was decreased as well. Yeah, we had restrictions in the OT, and we cannot have too many people around when having bedside tutorials.”  P03 “Definitely less exposure to real patients and clinical signs in the wards”  P07 “I am not able to take a history with the patient themselves, and I think more importantly, the visuals are also quite limited and also not being able to appreciate the physicals signs of classical conditions”  P11 “What I lost in the pandemic due to the restrictions is not the knowledge, but the practical skills and the experiences of being immersed in the different situations.” |
|  |  | Increase in online learning | P05 “I think it was affected because our postings were cut short and a lot of lessons were moved online.”  P12 “The learning is shifted online, such as having more Zoom lectures. There is less practical aspect of learning.”  P13 “… you cannot go to the hospital anymore, and then you have to attend lectures online, … you do not get to see the clinical presentations.” |
|  |  | Decreased clinical exposure | P04 “… since ward time has been reduced, I think there has also been a reduction in content delivery like bedside tutorials.”  P06 “… patient exposure was definitely reduced because we were not allowed to cross ward... Also, I think the duration of clinical postings shortened”  P11 “I think a lot of like opportunities were reduced and there were a lot more restrictions. So, it was more difficult to get practical hands-on learning.” |
|  |  |  |  |
| Theme 2: The role of mobile learning in medical education | | | |
|  | Mobile apps for learning in medical school | Study apps | P03 “For educational videos, I use the internet, YouTube, Osmosis and Amboss. For knowledge and practice questions, I use the internet, Telegram, and also other educational apps such as the flashcard app, Anki.”  P09 “My school uses Qstream which also prompts questions for your to answer ... There are also other applications that I use, such as Amboss which also does the same. I also use Anki.” |
|  |  | Messaging apps | P09 “I use the Telegram channels and as well as polls on Instagram stories.”  P12 “This Telegram group, some Instagram pages that our batchmates created, online notes and also YouTube.” |
|  |  | Preference for Telegram | P01 “Telegram has way more features like polling and asking questions and scheduling questions which are very useful for work purposes”  P01 “Telegram can work on the iPad but not WhatsApp”  P04 “Also, images come out in a much better quality. So, if there are case images, they also come out quite neat with regards to Telegram.”  P05 “Telegram is good because it has more features and has more stickers as well.”  P13 “I prefer Telegram because I think it is more user-friendly than WhatsApp and it is easier to navigate.” |
|  |  | Role of mobile apps | P02 “… it works to complement the main learning from like textbooks or seniors notes etc. and to reinforce whatever I have already learnt …”  P04 “Because it is virtual, there is a lack of face to face interaction and the ability to perform physical examination or take a proper face to face history. I think that is a big issue that people feel with regards to personnel competency in Medicine.” … “I think it is something that no amount of virtual learning can replace.”  P09 “… mobile-based education is more for contents while clinical skills have to come through the clinical context.” |
|  |  |  |  |
|  | Factors influencing the use of mobile apps | Free or affordable | P02 “For some mobile apps like Amboss, our school actually helped to pay for their subscription fees and so made it available for all of us to use.”  P09 “It is also nice that it is free.” |
|  |  | Useful or entertaining | P02 “if I think I learnt quite a lot from it or I feel that their way of teaching is effective, then I will use it more.” |
|  |  | Recommendations | P01 “Even my seniors who went through the rotations without the pandemic were already using them and recommended them to me”  P02 “It's more of a recommendation by word of mouth, like whether this particular app is good or not.” |
|  |  | Convenience and maximize time | P01 “These apps make learning accessible and convenient since we can study anytime and anywhere, even when queueing up for food or when travelling between places”  P03 “The greatest pull factor is how convenient it is to use the phone. I can easily use these apps when I am on public transport.”  P06 “We do not necessarily have to be in a classroom setting to complete the questions. We can complete it anywhere.” |
|  |  |  |  |
|  | Helpful features of mobile apps | Resources and information | P01 “There are also a lot of medical Telegram groups around which provide knowledge and information.”  P03 “They can be used by doctors or teachers to disseminate information to students or for students to ask questions to the doctors.”  P05 “The number of questions available on these question banks online is really a lot.”  P06 “Another pro is that it stimulates active recall.” |
|  |  | Two-way communication with tutors and peers | P01 “We also use these apps to share notes with each other.”  P02 “I think in my school, there are like, a few groups that me and my friends created, just to like practice taking histories from each other, or like, trying to identify what is an important topic and what is high yield to learn.”  P11 “I also use it to communicate with friends during postings and to receive posting information from official sources.” |
|  |  |  |  |
|  | Limitations of using mobile apps | Costs | P02 “Some apps require subscriptions or a one-time payment for use. So, such costs are like a barrier if you do not like to or do not wish to pay for these apps.” |
|  |  | Hardware limitations | P01 “The phone is also very small, so it is very difficult to edit documents on them.”  P01 “if I use my phone too much, the battery will also run out very quickly.”  P03 “it is harder for me to edit things because the phone keyboard is less versatile.” |
|  |  | Disorganization and unfriendly user-interface | P01 “I cannot transfer my notes from my phone to the computer easily.”  P01 “since I use these messaging apps for other purposes as well, these apps become more cluttered if I use them for learning purposes too.”  P01 “… it is very difficult to search for the different files because they will be like mixed with other non-work or -education related chats, which is very messy.”  P02 “some apps are difficult to use, especially when their user interface is very complicated and not user-friendly.”  P10 “It is also harder to take notes… It is harder to refer to notes or huge chunks of texts on the train as compared to when at home.” |
|  |  | Limited information on display | P05 “it is not as detailed as a textbook or something online which has more information.”  P10 “you cannot really put everything you are supposed to learn in a mobile application.”  P11 “An issue that I encounter with mobile learning, which also exists for hard-copy learning, is that explanations may not be complete.” |
|  |  | Poorly contextualized information | P13 “the conditions and presenting complaints are very localized to their countries. For example, in America, they have certain diseases that are more prevalent and to them, they are a must-know. But then to us, it is something that we were taught not to pay so much attention to.”  P13 “the reference values are all different, so it takes quite a bit of hassle to refer to the normal values and then go back to the question again.” |
|  |  | Distraction | P01 “I don't like it because questions and work messages get mixed together in one app and then it kind of distracts the learning. I will prefer a separate app if I want to use it for studying.” |
|  |  |  |  |
|  | TESLA to support surgical education | Augmentation of learning | P03 “It was especially useful when I was in other postings which did not allow me to encounter Surgical cases on a daily basis.”  P03 “I think it is quite useful because of the case-based style of questions. The question difficulty is appropriate …”  P05 “To be honest, I have not even seen before in real person some of the things in the group. So that was very useful for me to see and to learn.”  P09 “I found it quite useful, and I learnt a lot from it, especially because Surgery was my last posting, and I joined the group before that.”  P10 “The Telegram group is definitely a very useful tool to augment learning. Any form of revision questions will definitely help students in clarifying questions” |
|  |  | Credibility of content | P02 “the information in the group is provided by doctors and so, the information is more credible. Compared to the other groups, I would be more inclined to trust the information from this group.”  P10 “Since it is a channel created by a doctor, it makes the questions more legitimate as compared to if being made by students.”  P12 “the questions are generated by a doctor, which increases the validity and trust in the questions.” |
|  |  | Good quality of questions, explanations and supplementary material | P01 “the questions that the General Surgery MedEd telegram group provide are more clinically based and require more thinking instead of simply recalling a fact.”  P03 “The question difficulty is appropriate, and it does not test too esoteric stuff.”  P03 “the content posted is very relevant to the Singapore medical student, and it is the important stuff that you cannot miss.”  P05 “Some of the pictures are what students might not get the opportunity to see. So, I think that is good.”  P11 “I think the explanations provided are always very solid and consistent.” |
|  |  | Answer questions anonymously | P04 “It also allows you to have some anonymity when you answer so that you do not have the pressure of having to get it right. Instead, you can just put the best answer that you can think of.”  P09 “when it is an anonymous poll, it takes away any shame in answering the questions wrongly. Even if I do not know, I can just give it a shot and see how I am doing.” |
|  |  | Lack of anonymity when asking questions | P01 “I think it is weird that everyone in the group will be reading your question. So, I guess, maybe one thing that I would prefer is to ask questions anonymously.”  P04 “students may not really know what is the level of questions that they should be posting because they if they feel like they are asking very basic questions, it feels very silly. But if they ask a very complicated question, then they might not even be able to receive a very detailed answer over text which can be better articulated over a face-to-face setting. So, I think there is not a very clear indication on what is a good level to pitch a question.”  P10 “If it cannot be traced back to me, there is nothing for me to lose and I will just ask questions if I have doubts. Even if no one replies, then I will just forget about it. So, there is no harm trying.”  P10 “It seems that no one uses the comment feature. Probably it is because it is not anonymous, and people are shy to use it even if they have questions.” |
|  |  | Disorganization | P01 “I feel that the Telegram group is a bit messy as the questions are random ... Telegram is not able to provide such clear classification of the questions and a new learner might feel that their learning would be all over the place.”  P05 “I think now it is quite haphazard and not really organized and structured.”  P10 “it is hard to access previous questions. For example, if the group was created one year ago, you literally have to scroll all the way up to find a particular question.”  P10 “If you attempted some questions but with some in between, then it is very hard to find those that you have not done yet.”  P11 “If the content is posted in terms of links and there is an archive with a proper order, it might then be easier to search for the information.” |
|  |  | Word limit resulting in short explanations | P01 “the explanations are very short since there is a word limit to the questions and explanations. Hence sometimes I do not fully understand the explanation because it's not complete.”  P03 “What I have seen other channels do is that they send a short explanation as part of the poll first and then a longer one later.”  P07 “Sometimes there are questions in the Telegram group that are completely left without an explanation”  P08 “maybe when radiology pictures are being sent, it would help if the abnormalities can be pointed out in the explanations. Sometimes I look at the pictures and I do not know what I am looking at.”  P10 “Providing justification is the most important point as if there is no justification, even with the correct answer, there is no point, and it does not help the learning as much as you can only memorize the answers.”  P12 “I think the explanations to the questions are quite short. Hence, I think there is room to expand on that, for instance providing resources for students on where to find articles or guidelines. Maybe the doctors can also explain their approach to solving the questions. It would help lead to a more comprehensive understanding of the explanations.” |
