# Supplementary material for: The Use of Telegram in Surgical Education: Exploratory Study
Source: JMIR Med Educ. 2022 Sep 27;8(3):e35983. doi: 10.2196/35983 (PMC9518707; doi:10.2196/35983)
Supplement: Multimedia Appendix 3 [file mededu_v8i3e35983_app3.docx]

Appendix 3: COREQ 32-item checklist^1^

| **Item** | **Guide questions/description** | **Reported on page (#)** |
| --- | --- | --- |
| Domain 1: Research team and reflexivity | | |
| 1. Interviewer/facilitator | Which author/s conducted the interview? | 6 |
| 1. Credentials | What were the researchers’ credentials? | 1 |
| 1. Occupation | What was their occupation at the time of the study? | 6 |
| 1. Gender | Was the researcher male or female? | 6 |
| 1. Experience and training | What experience or training did the researcher have? | 6 |
| 1. Relationship with participants established | Was a relationship established prior to study commencement? | No |
| 1. Participant knowledge of the interviewer | What did the participants know about the researcher? | None |
| 1. Interviewer characteristics | What characteristics were reported about the interviewer/facilitator? | 6 |
| Domain 2: Study Design | | |
| 1. Methodological orientation and theory | What methodology orientation was started to underpin the study? | 4-6 |
| 1. Sampling | How were participants selected? | 5 |
| 1. Method of approach | How were participants approached? | 5 |
| 1. Sample size | How many participants were in the study? | 6 |
| 1. Non-participation | How many people refused to participate or dropped out? Reasons? | None |
| 1. Setting of data collection | Where was the data collected? | 6 |
| 1. Presence of non-participants | Was anyone else present besides the participants and researchers? | No |
| 1. Description of sample | What are the important characteristics of the sample? | 6 |
| 1. Interview guide | Were questions, prompts, guides provided by the authors? | 5 |
| 1. Repeat interviews | Were repeat interviews carried out? | No |
| 1. Audio/visual recording | Did the research use audio or visual recording to collect the data? | 6 |
| 1. Field notes | Were field notes made during and/or after the interview? | No |
| 1. Duration | What was the duration of the interviews? | 6 |
| 1. Data saturation | Was data saturation discussed? | 5 |
| 1. Transcripts returned | Were transcripts returned to participants for comment and/or correction? | No |
| Domain 3: analysis and findings | | |
| 1. Number of data coders | How many data coders coded the data? | 6 |
| 1. Description of the coding tree | Did authors provide a description of the coding tree? | 6, Appendix 2 |
| 1. Derivation of themes | Were themes identified in advance or derived from the data? | 6 |
| 1. Software | What software, if applicable, was used to manage the data? | 6 |
| 1. Participant checking | Did participants provide feedback on the findings? | No |
| 1. Quotations presented | Were participant quotations presented to illustrate the themes/findings? Was each quotation identified? | 7-12, Appendix 2 |
| 1. Data and findings consistent | Was there consistency between the data presented and the findings? | 12-14 |
| 1. Clarity of major themes | Were major themes clearly presented in the findings? | 7-12 |
| 1. Clarity of minor themes | Is there a description of diverse cases or discussion of minor themes? | 7-12 |

1. Tong A, Sainsbury P, Craig J. Consolidated Criteria for Reporting Qualitative Research (COREQ): A 32-Item Checklist for Interviews and Focus Groups. International journal for quality in health care : journal of the International Society for Quality in Health Care / ISQua. 2008;19:349-57.
